# Properties of Surface-Active Organics in Aerosol Particles Produced from Combustion of Biomass Fuels under Simulated Prescribed-Fire and Wildfire Conditions

**DOI:** 10.1021/acsestair.4c00243

**Published:** 2025-01-03

**Authors:** Ariana M. Deegan, Chase K. Glenn, Omar El Hajj, Anita Anosike, Kruthika Kumar, Muhammad Abdurrahman, Bin Bai, Pengfei Liu, Joseph O’Brien, Rawad Saleh, Amanda A. Frossard

**Affiliations:** † Department of Chemistry, University of Georgia, Athens, Georgia 30602, United States; ‡ School of Environmental, Civil, Agricultural and Mechanical Engineering, University of Georgia, Athens, Georgia 30602, United States; § School of Earth and Atmospheric Sciences, Georgia Institute of Technology, Atlanta, Georgia 30332, United States; ∥ U.S. Department of Agriculture Forest Service, Athens, Georgia 30602, United States

**Keywords:** biomass burning, organic aerosol, surface tension, surfactants, wildfire, prescribed fire

## Abstract

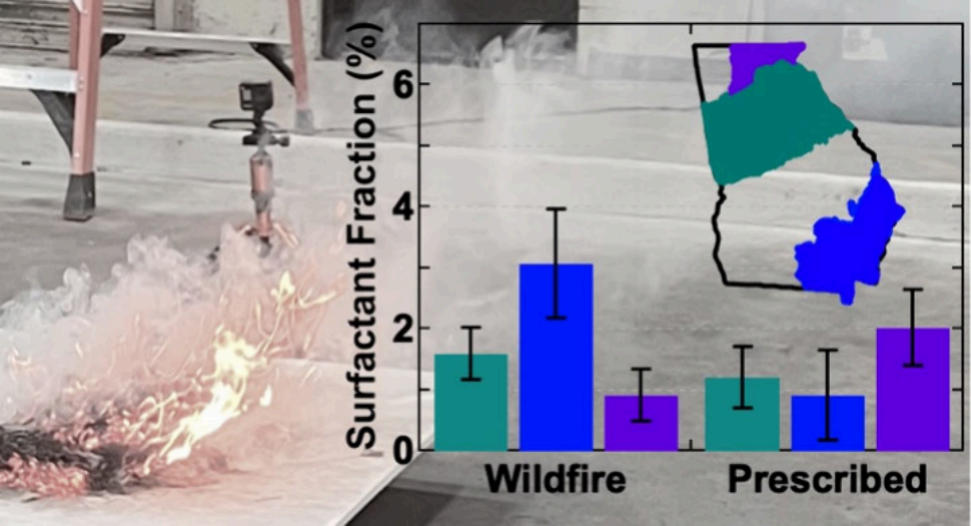

The interfacial properties of the organic fraction of biomass burning aerosols (BBA), such as the critical micelle concentration (CMC) and surfactant composition, may vary based on the origin and moisture content of the fuel and the resulting combustion conditions. Surfactant composition, fraction of total particle mass, surface tension minimums, and CMC values of organics extracted from fresh and aged BBA produced using fuel beds from Georgia ecoregions (Piedmont, Coastal Plain, and Blue Ridge) and with fuel moisture contents representative of prescribed fires or drought-induced wildfires were measured using high resolution mass spectrometry, UV–vis spectroscopy, and pendant drop tensiometry. Surface tension minimums of organics extracted from all BBA were low (<45 mN m^–1^), and surfactants were ∼2% of the total particle mass. The surfactant fraction was tied to combustion conditions, with the highest fractions present in BBA produced from the most efficient (highest temperature) combustion. Aging of BBA using a potential aerosol mass oxidative flow reactor resulted in an increase in the surfactant fractions of total BBA mass. The dependence of the surfactant fraction on combustion conditions may have implications for the microphysics of BBA from wildfires and prescribed fires.

## Abstract 2

Understanding the chemical and physical characteristics of surfactants in biomass burning aerosols is important for understanding their influence on the climate. The concentration, composition, and surface tension of surface-active organics in biomass burning aerosol varied based on the fuel-bed composition and moisture content.

## 1.0 Introduction

Biomass burning aerosols (BBA) comprise a large portion of atmospheric aerosols, contributing 75% of global, organic aerosol emissions annually^1^ and modulate the climate through direct absorption and scattering of solar radiation and through modification of aerosol–cloud interactions.^2−4^ The composition of BBA ranges with different classes of carbonaceous particulate matter, and the highest uncertainties of the influence of BBA on radiative forcing lie in the composition and properties of the organic fraction, which contains brown carbon compounds characterized by their absorption in the UV and visible range.^5,6^ The difficulty in characterizing and quantifying organics in BBA is attributed to differences in overall composition and concentrations of particles produced from burning events whose emissions vary based on the composition and moisture content of the fuel, resulting in different combustion conditions, as well as atmospheric aging.^5,7−9^ Studies have emphasized the importance of regional source profiles for biomass burning emissions using different fuels.^10^ The moisture content of fuels in burning events have been shown to influence the efficiency of combustion and the resulting composition of the emissions.^5,11^ Measuring the differences in BBA composition and physical properties based on different combustion conditions will increase our understanding of their environmental impact.

The water-soluble organic carbon fraction of BBA has shown enhanced surface activity,^5,12,13^ affecting its cloud condensation nuclei (CCN) potential and consequently its indirect climate impact.^14^ Previous studies have attributed the CCN activity of BBA from smoldering to the water-soluble organic fraction, which is more hygroscopic than the water-insoluble organic fraction.^15,16^ Other studies saw varying CCN activity for particles produced from different burn conditions.^17^ Giordano et al.^18^ reported that BBA depressed surface tension by ∼30% or more compared to that of pure water (surface tension depressions were calculated as the percent difference of the surface tension of the BBA solution and that of pure water, measured using pendant drop tensiometry). The study also observed a factor of 2 decrease in the hygroscopicity parameter (κ) calculated from measurements of CCN activity when experimentally measured surface tension values were applied compared to those calculated using the surface tension of pure water.^18^

Surface-active organics may decrease the surface tension of atmospheric aerosols, depending on factors such as droplet size and composition,^19−22^ and may thus lower the energy barrier of cloud activation, leading to increased CCN potential.^23−25^ Surfactants are a class of surface-active organics that have previously been measured in ambient aerosols^26−29^ at total atmospheric particle mass fractions of 1.5 to 7%.^27,28^ Solutions of surfactants extracted from atmospheric aerosol have been found to contribute to surface tension depression in diluted aqueous droplets, measured with pendant drop tensiometry.^27,28^ Their structure consists of a hydrophilic polar headgroup and hydrophobic alkyl chain that drives their adsorption to the surface of solutions, decreases surface tension at an interface, and causes self-aggregation into micelles.^30,31^ The critical micelle concentration (CMC) of the surfactant mixture within a particle can influence the particle physicochemistry.^32−34^ Specific to individual surfactants, the CMC is the point where surfactant monomers begin to self-aggregate into micelles and no additional surface tension depression occurs.^31,35^ The charge of the surfactant, the number of carbons on the alkyl chain, functional groups, chemical structure, and the addition of ions in solution influence the CMC.^26,31,35^ The composition, concentration, and physical properties of surfactants in BBA have not been heavily studied and require further investigation as a potential contributors to CCN activity.

Surface tension depression in BBA has previously been attributed to surface-active compounds known as humic-like substances (HULIS), which are named for their similarity in structure and optical properties to macromolecular organic humic substances consisting of humic acid, fulvic acid, and humin.^33,36,37^ The chemical composition of HULIS varies greatly and consists of smaller aliphatic and aromatic compounds containing different functional groups, such as carboxyl, hydroxyl, and carbonyl groups.^37,38^ BBA and environmental humic substances have previously been observed to have surface tension depressions of 20–42%, compared to surface tensions of pure water,^14,18,39,40^ and other studies have observed surface tension minimums measured with pendant drop tensiometry to be 41.4 to 49.6 mN m^–1^ in HULIS extracts.^39,41^ HULIS observed in atmospheric aerosol particles has shown enhanced surface activity and structures with lower aromaticity and lower molecular weight, compared to terrestrial and marine HULIS.^33^ Additionally, HULIS has been attributed to increased CCN activity in BBA.^42,43^ Dinar et al.^44^ determined that HULIS extracted from fresh and aged smoke were more CCN active than extracted Suwannee River Fulvic Acid, which they considered as a proxy for atmospheric HULIS. While the focus of investigating surface tension depression in BBA has been on characterizing and quantifying HULIS compounds, a targeted approach to different classes of surface-active organics and their surfactant-like properties may provide greater insight into the contribution of organics in BBA to CCN activity.

In this study, the surfactant mass fraction, ionic composition, surface tension, and CMCs of surface-active organics in BBA collected from biomass burning experiments were measured during the Georgia Wildland-fire Simulation Experiment (G-WISE). Here, we investigate how these surfactant properties vary as a function of fuel-bed composition, with fuels from different Georgia ecoregions and moisture contents reflective of wildfire and prescribed-fire conditions as well as atmospheric aging.

## 2.0 Methods

### 2.1 Experimental Overview

In this study, aerosol particles were produced, collected, and characterized as part of the Georgia Wildland-fire Simulation Experiment (G-WISE), a collaborative laboratory campaign conducted at the U.S. Forest Service Southern Research Combustion Facility in Athens, GA. Combustion experiments were conducted in a 1000 m^3^ burn room^45^ with fuel beds constructed using fuels collected from three different ecoregions in Georgia (Piedmont (P), Coastal Plain (CP), and Blue Ridge (BR)). The P and CP fuel beds contained only surface fuels, including woody and fine fuels. Woody fuels made up 18–20% of the total dry fuel mass and fine fuels made up 50–55% of the total dry fuel mass, with additional surface fuels containing other leaves and grasses that made up 24–32% of total dry fuel mass.^45^ In addition to surface fuels, the BR fuel bed also contained duff, which constituted more than 89% of the dry mass, and the woody fuels constituted 3–4% of the dry mass.^45^ The remaining dry fuel mass of the BR fuel-bed consisted of leaves and grasses. More information on the fuel beds is described by Glenn et al.^45^

In addition to the three fuel ecoregions that make up the fuel beds, two fuel moisture contents were investigated to model commonly observed burn conditions. Fuel moisture content is the amount of water in a fuel as a fraction of the dry weight. Fuel moisture content of each fuel was measured using a Model MAX 4000XL – Moisture and Solids Analyzer (Computrac), after conditioning the fuel by drying, wetting, or humidifying, as described for this study by McQueen et al.^46^ Low fuel moisture content included fuels conditioned to <3% to represent the fuel moisture during drought-induced wildfires (referred to here as “Wild” and “low fuel moisture content”). High fuel moisture content included fuels conditioned to 10% and 32–50% for the fine and woody fuels, respectively, to represent moisture contents typically encountered in prescribed fires (referred to here as “Rx” and “high fuel moisture content”). The duff in the BR fuel beds was dried to <3% moisture content for Wild and was used as collected from the field for Rx (moisture content of 50–60%). As described by Glenn et al.,^45^ the difference in moisture content between Rx and Wild leads to differences in combustion conditions. These combustion conditions are further discussed in Section 3.1.

For each experiment, after the conclusion of the burn, the valves were opened to allow smoke from the burn room to be collected and characterized. Each set of burn conditions were performed three times for reproducibility. To compare the effect of particle aging, the smoke was aged for select experiments using a Potential Aerosol Mass Oxidation Flow Reactor (PAM OFR; Aerodyne Research, Inc.).^47^ Here, we discuss aging from two experiments using fuel beds with fuel from the CP ecoregion and both fuel moisture contents. Particles were aged using OH oxidation^47,48^ at a residence time of 105 s.^48^ The OH exposures, as functions of concentration (molecules per cm^3^) and time (s), were estimated as 1.52 × 10^12^ molecules cm^–3^ s for CP-Rx and 7.91 × 10^11^ molecules cm^–3^ s for CP-Wild. We note that the initial aerosol mass concentrations in the burn room (after completion of the burns) were on the order of several mg m^–3^ (Table 1), which is too high for sampling through the OFR and the online instruments deployed during G-WISE. Therefore, prior to collecting the aged filter samples, the smoke in the burn room was vented to reduce the aerosol mass concentrations. We also note that while we used the PAM OFR to simulate particle aging in the atmosphere, this is just one mechanism by which particles may be aged and may not represent all atmospherically relevant aging and conditions in the atmosphere.

**Table 1:** Averages and Standard Deviations of Inorganic Percents of Total Particle Mass (PM), nmols of Surfactants, Total PM, Surfactant Mass, Surfactant Percents of Total PM, Surface Tension Minimums (ST min), and CMC Values for All Fuel-Bed Types

	P-Wild	P-Rx	CP-Wild	CP-Aged Wild	CP-Rx	CP-Aged Rx	BR-Wild	BR-Rx
No. of samples	2	3	3	1	3	1	3	3
FRE_norm_^a^ (MJ/kg)	1.31 ± 0.40	0.82 ± 0.02	1.38 ± 0.14	N/A	0.74 ± 0.05	N/A	0.50 ± 0.05	0.70 ± 0.05
NH_4_^+^ (% of PM)	BB^c^	0.64	BB^c^	0.27	1.96	0.82	0.44	BB^c^
K^+^ (% of PM)	0.31	0.24 ± 0.03	0.30	0.01	0.39 ± 0.18	0.01	BB^c^	BB^c^
SO_4_^2-^ (% of PM)	19.24	16.15 ± 3.65	20.31 ± 9.51	BB^c^	11.30 ± 13.75	0.21	0.18 ± 0.06	0.13
NO_3_^–^ (% of PM)	0.39	0.54 ± 0.49	0.91 ± 0.89	BB^c^	0.05	0.57	BB^c^	BB^c^
surfactant amount (nmol)	23.9 ± 2.2	20.3 ± 8.7	35.8 ± 14.7	20.7	15.5 ± 12.5	15.1	26.7 ± 8.2	15.5 ± 3.7
surfactant mass (μg)	7.17 ± 0.65	6.09 ± 2.60	10.73 ± 4.41	6.21	4.66 ± 1.07	4.54	7.99 ± 2.47	4.64 ± 1.10
PM mass (μg)	462 ± 82	502 ± 18	348 ± 83	80	505 ± 47	134	918 ± 164	233 ± 16
surfactant (% of PM)	1.59 ± 0.42	1.21 ± 0.51	3.08 ± 0.90	7.76	0.91 ± 0.75	3.39	0.91 ± 0.43	2.01 ± 0.62
ENVI-18 ST min (mN m^–1^)^b^	42.1 ± 6.4	39.6 ± 2.7	35.4 ± 1.1	43.5 ± 0.3	34.3 ± 0.4	43.1 ± 0.6	36.0 ± 3.0	37.2 ± 2.2
ENVI-Carb ST min^b^ (mN m^–1^)	59.3 ± 1.7	54.4 ± 7.3	49.9 ± 13.2	49.1 ± 1.0	42.5 ± 9.8	62.9 ± 0.8	44.5 ± 11.9	43.0 ± 7.3
ENVI-18 CMC (mM)	NC^d^	0.14	0.43	NC^d^	0.22	NC^d^	0.08	0.05
ENVI-Carb CMC (mM)	NC^d^	NC^d^	0.18	NC^d^	0.06	NC^d^	0.19	0.13

a Normalized fire radiative energy (FRE_norm_) averaged from values reported by Glenn et al.^45^

b Standard deviations calculated from repeated measurements for the same sample.

c BB indicates values that were below the blank and thus less than 0 after blank subtraction.

d NC designates surface tension curves from which CMCs could not be calculated.

### 2.2 Particle Collection

For this study, aerosol particles were collected on 47 mm prebaked quartz fiber filters (Pall Life Sciences) using polyethylene bulk impactors, with no particle diameter size cut, through 1/4″ copper tubing at a flow rate of 4.7 L min^–1^. Fresh particles were collected directly from the burn room for 30 min, while aged particles were collected through the PAM OFR for ∼17 h. Following collection, filters were immediately placed into precleaned 6-dram glass vials, capped, wrapped in foil, and stored frozen until analysis.

Particle filter blanks were collected by loading the prebaked quartz fiber filters into the holders and removing them after 5 min with zero flow. Blank samples were collected prior to the ignition of the burns. Blank samples were processed and analyzed the same as the samples.

To determine the total aerosol mass collected on the filters, the dry number size distributions of particles were measured using a scanning mobility particle sizer (SMPS, TSI), with an upstream drier, for particles with mobility diameters from 10 nm to 1 μm, as described by Glenn et al.^45^ An assumed density of water (1000 kg m^–3^) was used in the conversion to aerosol mass (μg m^–3^).^45^ Size distributions of supermicron particles were not measured in this study, since most of the particle number was assumed to be submicron, based on previous BBA studies.^49,50^

Fresh BBA were aged in the PAM OFR by turning on the UV lights, and aerosol size distributions were measured for both the fresh and aged BBA using the SMPS. These distributions were then integrated to obtain concentration measurements every 90 s. An exponential decay model was fit to the fresh BBA concentrations to account for losses in the burn room. The difference between the fresh and aged BBA mass concentrations represents the enhanced organic aerosol mass concentration and is thus referred to as the secondary organic aerosol mass concentration.

### 2.3 Particle Extraction and Separation of Large Organics

The general method first includes the extraction of particles from the filters. Then, organic molecules are isolated using solid phase extractions. The organic molecules that complex with specific dyes are quantified and considered to be surfactants, based on the properties necessary for the complexation. Surfactants are further confirmed based on the low surface tension of the organic extracts compared to that of pure water. Previous studies using this method have also confirmed the likelihood of surfactants using mass spectrometry.^51^

Particles were extracted from filters using previously described methods, which are explained briefly here.^52−54^ For each sample, 5 mL of ultrapure water was added to each glass vial containing an individual quartz fiber filter. The solution was vortexed in the vial for 10 min, sonicated for 10 min, and then filtered using a 0.45 μm polyether sulfone membrane syringe filter (VWR) into an additional precleaned glass vial. Each filter underwent two extractions, resulting in a total particle sample extract volume of 10 mL.

Large organic molecules were then extracted using two solid phase extraction cartridges, in series ENVI-18 (C18 sorbent material, 0.5 g bed weight, MilliporeSigma) and ENVI-Carb (graphitized carbon sorbent material, 0.5 g bed weight, Millipore Sigma). Extractions using ENVI-18 cartridges have previously been shown to exhibit high extraction efficiencies for anionic and nonionic surfactants, while extractions using ENVI-Carb cartridges have been shown to exhibit high extraction efficiencies for cationic surfactants.^55^ This extraction method was previously described by Burdette et al.^51^ and is briefly discussed here. First, the ENVI-18 cartridges were conditioned using 6 mL of acetonitrile (MilliporeSigma) and then rinsed with 12 mL of ultrapure water. Then, the 10 mL particle extract solutions were processed through the ENVI-18 cartridge without vacuum. The unretained solutions were collected in precleaned vials and saved for further extraction. The ENVI-Carb cartridges were conditioned using 6 mL of acetonitrile (MilliporeSigma) and then rinsed with 12 mL of ultrapure water. The ∼10 mL solutions from the ENVI-18 extraction were then processed through the ENVI-Carb cartridges without vacuum. The unretained solutions were then collected in additional precleaned glass vials for further analyses with ion chromatography. The cartridges were dried with vacuum, and 4 mL of acetonitrile was used to extract the organic fractions from the sorbent material in each of the cartridge pairs, individually, into precleaned glass vials. The acetonitrile was evaporated using nitrogen gas (99.998% purity, Airgas), and the dried organic extracts in the vials were stored at 4 °C for analysis with tensiometry, UV–vis spectroscopy, and mass spectrometry.

### 2.4 Major Ion Measurement with Ion Chromatography

The concentrations of select major ions in the particle samples were measured using two Dionex Integrion high-pressure ion chromatography (Thermo Fischer Scientific) instruments, as described by Burdette et al.^51^ Samples and blanks were prepared by filling ion chromatography vials with 1 mL of the ∼10 mL final nonretained solutions from the solid phase extractions and 4 mL of ultrapure water. The autosampler split the 5 mL volume between the two instruments, with one column quantifying the cations (Thermo Fischer Scientific) and the other quantifying the anions (Thermo Fischer Scientific). For this study, we targeted sodium, ammonium, potassium, chloride, sulfate, and nitrate, since those were previously reported in a BBA study.^56^ The concentrations of the corresponding sample blanks were subtracted from the initial sample concentrations. Concentrations in solution were calculated from calibration curves for each of the ions of interest. Sodium concentrations were higher than expected in the blank and may have been due to contamination. Chloride concentrations were all less than those in the blank. So here, we report the concentrations of cations ammonium and potassium and anions sulfate and nitrate in each solution, which were above the detection limits, above the blanks, and previously observed in BBA.^56^ Ion concentrations in particle samples were converted to mass values (in μg) by using the molecular weight of the ions. Total fractions of inorganic ions in the particle mass were calculated by dividing the ion mass by the total particle mass collected on the filter.

### 2.5 Surface Tension Analysis

Surface tension of the organic extracts was measured by using pendant drop tensiometry (Dataphysics). This method applies the deformation of a hanging droplet to the Young–Laplace equation to determine the surface tension of a solution.^57,58^

The dried organic extracts from the ENVI-18 and ENVI-Carb cartridges were each brought to room temperature and then rehydrated with 40 μL of ultrapure water. The samples each had 8 additional dilutions, ending in final volumes of 3840 μL, and the surface tension was measured at each step. Measurements with surface tension values less than and greater than 50 mN m^–1^ were completed using a 0.30 mm and 0.51 mm needle tip, respectively. The average droplet volumes measured using the 0.30 mm and 0.51 needle tip were 2.67 ± 0.57 and 5.40 ± 0.55 μL, respectively. Surface tensions of five droplets were measured at each dilution step, at 10 s intervals, following a 30 s equilibration.^41,59^ The equilibrium time allows the droplet to stabilize after dispensing and allows time for the surfactants to come to equilibrium within the droplet.^60,61^ The mean standard deviation of the droplet volume over the 10 s period was ±0.023 μL, indicating little to no evaporation of the droplets during the measurements.

The surface tension at the first point (40 μL) is here termed the surface tension minimum. To create the surface tension isotherms, the surface tension was plotted as a function of the log-normal surfactant concentration at each dilution step (described in Section 2.6). The CMC for one extract representing each experimental condition was calculated from the adsorption isotherm using procedures described previously.^27,28,62^ CMC calculations of standard surfactants using this procedure have shown good agreement with literature values.^27^ The CMC for each isotherm was calculated as the concentration at the intersection of two lines fit to the isotherm. The first line is horizontal and includes the minimum surface tension, and the second is the regression line fit to at least three points in the declining slope of the isotherm.^27^ CMCs were not calculated for isotherms that did not exhibit the presence of a horizontal plateau near the surface tension minimum or immediately increased in surface tension going from the minimum surface tension to the next dilution.

### 2.6 Colorimetry Analysis for Surfactant Quantification

The concentrations of the three ionic classes of surfactants (anionic, cationic, and nonionic) were measured in the organic extracts using colorimetry and UV–vis spectroscopy. This method has been used in previous studies for quantification of surfactants in ambient aerosol samples^27,54^ and is described briefly here. The dried organic fraction extracted with the ENVI-18 and ENVI-Carb extractions were hydrated to 3840 μL for surface tension measurements, as described in Section 2.5. The dissolved extracts were each separated into three aliquots and mixed with dyes specific to the targeted organic class: ethyl violet, disulfine blue, and cobalt thiocyanate for anionic, cationic, and nonionic surfactants, respectively. The dye–surfactant complexes were extracted in specific organic solvents: toluene for anionic surfactants and chloroform for nonionic and cationic surfactants. The absorption of the organic phase containing the surfactant–dye complex was measured by using a Cary 60 UV–vis spectrometer (Agilent Technologies). The absorbance values at 612, 628, and 317 nm were used to calculate the molar concentration of anionic, cationic, and nonionic surfactants, respectively, in each solution, using corresponding calibration curves. Calibration curves were created from surfactant standards sodium dodecyl sulfate (SDS) and dioctyl sulfosuccinate sodium salt (AOT) for anionic surfactants, cetyl triammonium chloride (CTAC) and Hyamine for cationic surfactants, and Brij 35 for nonionic surfactants.

The expected absorption of the nonionic surfactant-dye complex at 317 nm overlapped with other absorption peaks in the range of 200–400 nm, which may be due to the presence of HULIS^33,63^ and other brown carbon compounds^5,64,65^ in the solid phase extracts that were extracted into the organic phase but not bound with a specific dye. While this may have been occurring with the cationic and anionic surfactant analyses as well, the peak absorbances of their dyes did not fall in the 200–400 nm range impacted by HULIS and brown carbon. Because we could not definitively separate the HULIS absorption from that of the nonionic surfactant-dye complex, and to avoid overestimating the concentration of nonionic surfactants, they are excluded from this discussion.

Total surfactant concentrations are reported as the sum of anionic and cationic surfactant concentrations. Because nonionic surfactants are excluded from this sum, as mentioned in the previous paragraph, the total surfactant concentrations reported herein are considered to be the lower bounds. Extraction efficiencies were calculated for these measurements, as described in text S1, and applied to the anionic and cationic measured concentrations. The anionic surfactant extraction efficiency for the ENVI-18 cartridge was 63% (Figure S1), and the cationic surfactant extraction efficiency for the ENVI-Carb cartridge was 19% (Figure S2). Mean detection limits were calculated following the method in Frossard et al.^62^ and Keene et al.^66^ and were found to be 1.75 and 0.36 nmols per sample for anionic and cationic surfactants, respectively. This method of determining mean detection limits uses the standard deviation of the moles of surfactants measured from the blank samples and the particle extract volume (mL).

Surfactant concentrations are reported in nmol per m^–3^ of sampled air and were calculated from the measured moles of surfactant molecules per filter divided by the total volume of air sampled. To determine the contribution of surfactants to the total particle mass, the molar concentrations of surfactants were converted to mass concentrations in μg m^–3^ by using an assumed molecular weight of 300 g mol^–1^, consistent with the molecular weight of HULIS and surfactants from BBOA.^14,33^ To account for the differences in particle numbers and masses produced in different experiments, surfactant mass was normalized to total particle mass. Surfactant fraction was calculated as the surfactant mass divided by the total particle mass collected on the filter.

### 2.7 High Resolution Mass Spectrometry

Two sets of organic extracts from BBA produced using fuel from the CP ecoregion and the Wild and Rx moisture conditions were selected for further chemical analysis by mass spectrometry. The samples underwent the same particle and solid phase extractions described in previous sections. The organic extracts were then rehydrated in 1 mL of 1:1 ultrapure water and methanol solution and analyzed using an electrospray ionization quadrupole time-of-flight mass spectrometer (ESI-Q-TOF-MS; Impact II, Bruker) at the Proteomics and Mass Spectrometry (PAMS) facility at UGA. Further description of the instrument parameters can be found in Burdette et al.^67^ and briefly here. Organic extracts from ENVI-18 extractions were analyzed in negative ionization mode, while ENVI-Carb extracts were analyzed in positive ionization mode. A methanol carrier at a flow rate of 160 μL/h was used, and spectra were collected in the 50–1500 Da mass to charge range.

Formulas were assigned using a custom MATLAB code^68^ using a mass tolerance of 1 ppm. Chemical formulas were assigned based on elemental ranges previously observed for both surfactant^67^ and HULIS extracts:^38,69^ C_5–50_, H_1–200_, O_0–30_, N_0–5_, and S_0–2_. Only formulas that were unambiguously assigned to a specific peak are discussed in this study.

## 3.0 Results

### 3.1 Physical and Chemical Properties of Fresh BBA

The overall production of aerosols during the biomass burning experiments varied with the fuel-bed composition and moisture content. The average total particle mass loadings collected onto the filters for the 30 min sampling period for each experiment is shown in Table 1 and ranged from 217 to 1095 μg for fresh particles. The peak in the number size distribution of the BBA was similar throughout the study and ranged from 157 to 322 nm, in dry diameter. Additionally, the peak in the mass size distribution was similar throughout, ranging from 310 to 480 nm.

Trends in combustion conditions for each experiment depended on the fuel-bed composition and moisture content. As described by Glenn et al.^45^ combustion conditions during this study were characterized using fire radiative energy normalized by the dry mass of available fuel (FRE_norm_). The average values for each experiment, as retrieved by Glenn et al.^45^ are listed in Table 1. Briefly, the FRE_norm_ represents the total amount of radiative heat released from a fire per unit mass of available fuel and is thus a measure of combustion efficiency. Higher FRE_norm_ is associated with more efficient combustion and consequently higher combustion temperatures (more flaming), whereas lower FRE_norm_ is associated with lower combustion temperatures (more smoldering). For fuel beds that contained surface fuels only (P and CP), FRE_norm_ in Rx was on average lower than that in Wild due to the higher moisture content in Rx. However, the existence of duff in the BR fuel bed had an opposite effect on FRE_norm_. Due to its high moisture content, duff did not ignite under Rx conditions, but did ignite under Wild conditions and exhibited prolonged smoldering.^45^ The highest FRE_norm_ (i.e., the highest combustion temperature and most flaming) was observed for the CP and P Wild burns (Table 1). The FRE_norm_ values for the Rx conditions were similar for all three fuel beds and less than those of the CP and P Wild. The BR-Wild had the lowest FRE_norm_ (i.e., the lowest combustion temperatures and most smoldering).^45^ Prescribed fires typically exclude duff ignition intentionally,^70^ but duff can burn in wildfires that occur after long periods of drought.^71^ Therefore, the difference in combustion conditions between the BR-Rx and BR-Wild burns in our experiments was representative of field observations.

The major ions identified in the fresh BBA particle extracts were ammonium, potassium, sulfate, and nitrate. The average fraction of major ions in the particles was 0.83, 0.23, 10.50, and 0.56% for ammonium, potassium, sulfate, and nitrate, respectively. Sulfate is the only major ion with a significant mass fraction in the BBA. The inorganic fraction did not vary greatly for the BBA produced from the CP and P fuel beds for both Wild and Rx. The sulfate fraction was the largest and varied between ∼80 to 97% of the measured inorganic mass. Consistent with our study, measurements of ambient biomass burning events show sulfate to be the largest fraction of particle mass,^72−74^ with ∼10% contributing to overall PM_2.5_ mass concentrations.^75,76^ However, the BBA from the BR fuel bed had a higher fraction of ammonium than sulfate in the Wild condition and only sulfate above the detection limit in the Rx condition. This difference in composition could be due to the presence of duff in the BR fuel bed, which altered the combustion conditions (Table 1). The smoldering conditions of BR-Wild may have caused the larger fraction of ammonium in that sample. Previous studies have observed variability in mass percentages of ionic species in ambient biomass burning aerosols based on vegetation types, geographical region, and combustion phase.^77^

### 3.2 Surface Tension of Organic Extracts and Corresponding CMCs

The minimum surface tensions of the organics extracted from the fresh BBA produced from the Rx and Wild moisture contents and fuels measured across all ecoregions ranged from 33.3 to 46.6 mN m^–1^ and from 28.2 to 61.5 mN m^–1^, using the ENVI-18 and ENVI-Carb cartridges, respectively (averages specific to each ecoregion and moisture condition are listed in Table 1). These values are lower than the surface tension of pure water (72.0 mN m^–1^)^78^ and consistent with previous values of minimum surface tensions of surfactants extracted from atmospheric aerosol particles using an extraction through a C-18 sorbent material (34.8 to 48.3 mN m^–1^).^51^ This demonstrates the presence of surfactants in all of the BBA samples and shows that surfactants are emitted during the combustion of different fuels under both the Wild and Rx fuel-bed moisture conditions. The different ranges for the two extracts demonstrate that different surfactants and/or different masses of surfactants were extracted from each. This is consistent with the ENVI-18 cartridges having high extraction efficiencies for anionic surfactants and the ENVI-Carb cartridges having high extraction efficiencies for cationic surfactants.^55^

**Figure 1:**
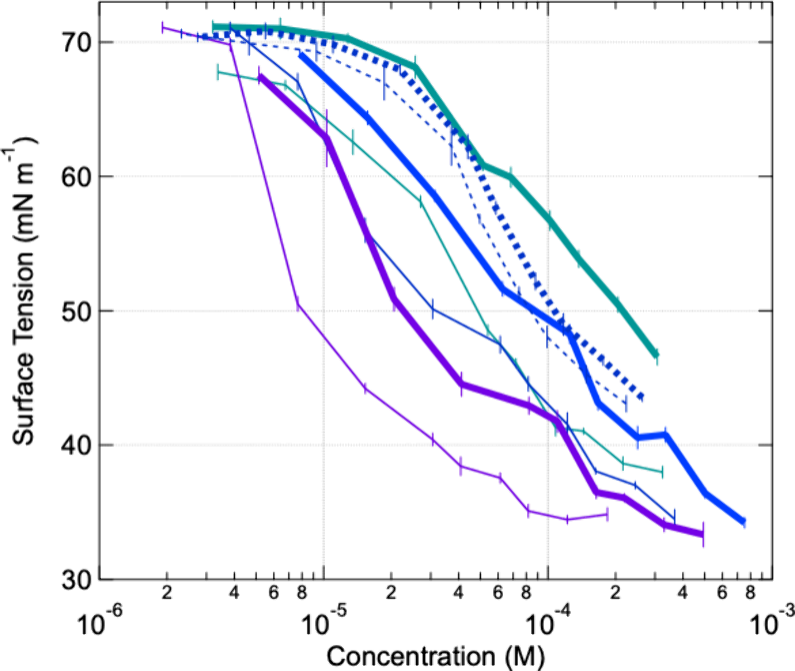
Surface tension isotherms for surfactants extracted using ENVI-18 cartridges from the aerosol produced from the combustion of the three fuel types, Piedmont (P; teal), Coastal Plain (CP; dark blue), and Blue Ridge (BR; purple), and two fuel moisture contents, Wild (thick lines) and Rx (thin lines). Dashed lines represent surface tension isotherms of surfactant extracts from aged BBA produced from the combustion of the CP fuel beds. Standard deviations shown are from the average of five measurements at each dilution step.

The CMCs for surfactants extracted from the fresh BBA produced from the Rx and Wild moisture contents and fuels from all ecoregions ranged from 0.05 to 0.43 mM, for both the ENVI-18 and ENVI-Carb extractions (Table 1). These CMC values are higher than those reported for biological surfactants measured in previous studies (0.003 to 0.2 mM).^27,79^ Here, only 9 of the 16 isotherms had shapes from which CMCs could be derived (Figure 1 and Figure S3). The CMCs presented here may be underestimated due to the exclusion of some CMCs that could not be calculated. This could contribute to an overall higher CMC in the actual BBA than that presented here. Additionally, atmospheric aerosols are a complex matrix of different surfactants and organics that may have different features, contributing to lower CMCs. The interaction between a nonionic and an ionic surfactant has been shown to decrease the CMC of an individual nonionic or ionic surfactants, while the interaction between surfactants with the same ionicity exhibit minimal change compared to their individual CMCs.^80,81^

**Figure 2:**
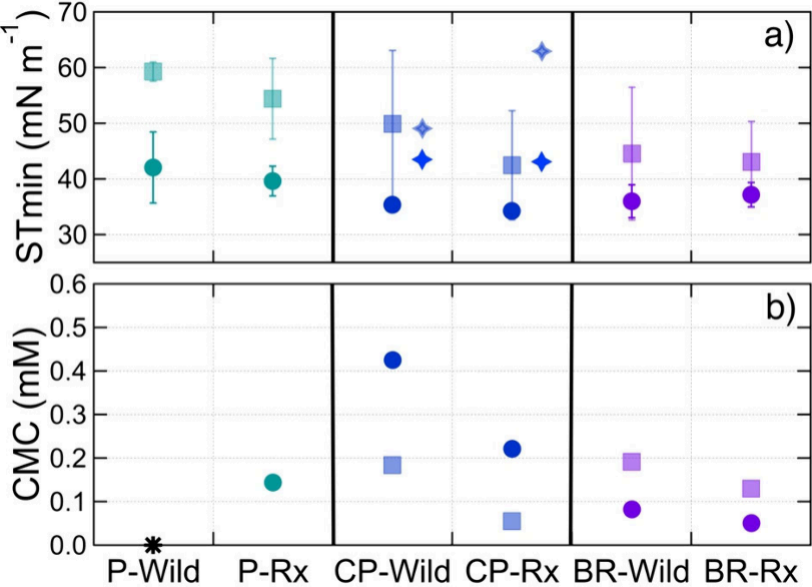
(a) Minimum surface tensions (STmin; mN m^–1^) and (b) critical micelle concentrations (CMC; mM) of the surfactants extracted using the ENVI-18 (circle) and ENVI-Carb (square) cartridges from the aerosol particles produced from the three fuel-bed types (Piedmont (P), Coastal Plain (CP), and Blue Ridge (BR)) and two fuel moisture contents (wildfire (Wild) and prescribed (Rx)). The dark and light diamonds for the CP ecoregion represent the STmin for surfactants from aged particles extracted using the ENVI-18 and ENVI-Carb cartridges, respectively. There were no calculated CMC values for the P-Wild sample extracts from both cartridges (indicated with asterisk) and no calculated CMC for the extract from the Envi-Carb cartridge for the P-Rx. Averages and standard deviations for the STmin are calculated from the replicate surface tension measurements.

The surfactants from the ENVI-18 extractions showed higher CMCs for the CP ecoregion under both moisture contents compared to the ENVI-Carb extractions; however, the opposite trend is shown for CMCs from the BR ecoregion, and only the CMC for the P-Rx was calculated for surfactants from the ENVI-18 extraction (Figure 2). This difference in CMC for ENVI-18 and ENVI-Carb extracts from different ecoregions may be due to the different extraction efficiencies of the target compounds in the two cartridges as well as the different potential mixture of surfactants in different ecoregions, including the mixture of nonionic surfactants with the ionic surfactants.

### 3.3 Mass Fraction of Surfactants in Fresh BBA

The total concentration of surfactants in fresh BBA from the three ecoregions and two moisture contents ranged from 12.8 to 370.5 nmol m^–3^, with a median of 156.3 nmol m^–3^. When using an assumed molecular weight of 300 g mol^–1^, which was observed at high peak intensities in the mass spectrum for the CP ecoregion fuel (Figure S4), the surfactant fraction of the total fresh particle mass for each ecoregion ranged from 0.09 to 3.65%. This is consistent with previous work that observed surfactants to be between 1.5 and 7.3% of the total particle mass of atmospheric aerosol particles from different regions.^28^ The lower averages reported herein could be because nonionic surfactants were not quantified in this study.

Surfactants measured in fresh BBA were majority anionic (average of 86% anionic and 14% cationic surfactants). Of the 20 samples analyzed, 18 had cationic surfactants above the mean detection limit. This composition of surfactants is consistent with previous studies, since HULIS are mainly anionic and make up a majority of the water-soluble organic carbon fraction in BBA.^33^ Additional studies have observed anionic surfactants to be the largest fraction of surfactants in atmospheric aerosol particles, with little to no presence of cationic surfactants.^27,28,82^

## 4.0 Discussion

### 4.1 Properties of Surfactants in BBA

#### 4.1.1 Variability in Surfactant Fraction Due to Fuel-Bed Composition and Moisture Content

**Figure 3:**
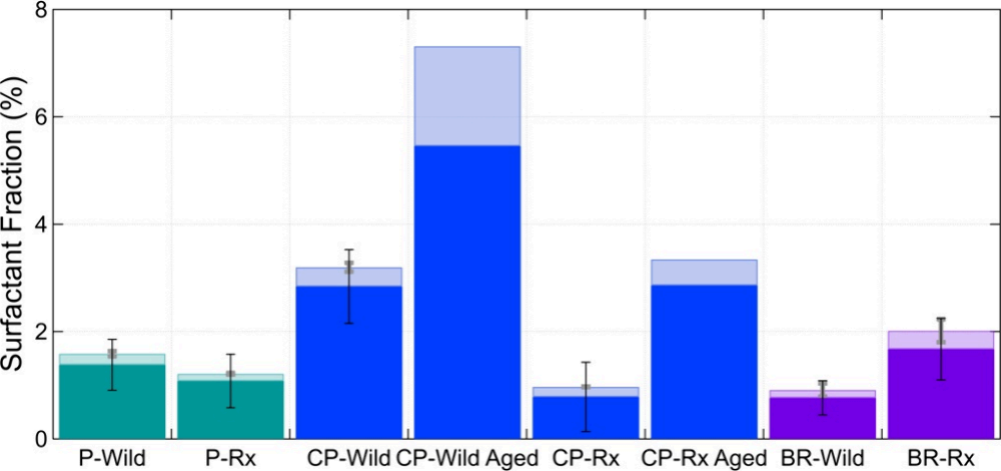
Surfactant fraction (mass of surfactants extracted from BBA divided by total particle mass) for BBA produced from the three fuel-bed compositions (Piedmont (P), Coastal Plain (CP), and Blue Ridge (BR)) and for both fuel moisture contents (wildfire (Wild) and prescribed (Rx)). Darker shades indicate anionic surfactants, and lighter shades indicate cationic surfactants. The thin black and thick gray error bars represent the standard deviations of the anionic and cationic surfactant fractions, respectively. The two aged bars were individual samples and thus have no standard deviations.

Organic extracts from fresh BBA produced from combustion of fuel beds with two moisture contents and from the three Georgia ecoregions exhibit variability in surfactant mass fractions and physical properties. The composition of the ecoregion fuels themselves exhibit differences based on the vegetation diversity from varying geographical regions within Georgia.^45^ The surfactant fractions of total PM for the BBA from the combustion of fuel beds from three ecoregions are also very similar and within the uncertainties when combined and averaged for both moisture fuel contents, with surfactants making up 1.4%, 2.0%, and 1.5% of the total PM for P, CP, and BR, respectively (averages for individual conditions are shown in Figure 3).

If compared individually, for the Wild moisture content, BBA from the CP fuel beds had the highest surfactant fraction of the total particle mass, while BBA from the BR fuel beds had the lowest surfactant fraction (Figure 3). The CP-Wild and P-Wild fuel beds had the same moisture contents and burned under relatively similar combustion conditions, as evidenced by the similar FRE_norm_ (Table 1). The difference in surfactant fraction of particle mass between these two thus indicates that the ecoregion from which the fuel bed was collected may also play a role in the resulting surfactant fraction. The BBA from the BR-Wild had the lowest mass fraction, consistent with the larger fraction of duff and thus the lower FRE_norm_ and associated lower combustion temperature and longer smoldering time.

For the Rx fuel moisture contents, the surfactant fractions were relatively similar for BBA from the CP and P fuel beds, but BBA from the BR fuel beds had the largest surfactant fraction. In the Rx conditions, the duff of the BR fuel beds did not burn; therefore, the only fuel that was combusted was fine and woody, similar to that of the CP and P fuel beds, and the Rx of all three fuel-bed types had similar combustion temperatures. This indicates that the ecoregion of the fuel bed may influence the resulting BBA surfactant fraction, similar to the Wild conditions.

Taken together, combustion conditions seem to have an effect on the production of surfactants. For fuel beds that contained surface fuels only (P and CP), the Wild burns emitted higher surfactant mass fractions compared to the Rx burns, indicating that the higher combustion temperatures in the Wild burns (and higher FRE_norm_) were more conducive to the formation of surfactants. The surfactant fraction of the BBA from BR-Rx is greater than that of BR-Wild, also consistent with the higher combustion temperature of BR-Rx compared to BR-Wild. The prolonged smoldering observed in the BR-Wild smoke experiments specific to the presence of duff contributed to greater incomplete combustion,^83^ which may have led to higher organic fractions, but did not lead to higher surfactant fractions. This may indicate that combustion temperature plays an important role in the emissions of organic compounds but that they may vary based on organic structure and properties.

While anionic surfactants made up the majority of the surfactant fraction of fresh BBA, the cationic fraction of total surfactants was higher in the BBA from the BR fuel beds (16%) compared to the P (13%) and CP (12%) fuel beds (Figure 3). The minimal variability of the cationic fraction of surfactants for the three fuel-bed types indicates that the fuel-bed composition did not significantly contribute to the differences in ionic composition (anionic vs cationic) of the surfactants. Averaging the Rx and Wild, the BR fuel beds had lower FRE_norm_ than the Rx and Wild of the P and CP fuel beds. This may demonstrate that the lower combustion temperatures in the BR fuel-beds produced more cationic surfactants, but the trends do not extend beyond the overall variability.

The surface tension minimums (measured as the surface tension of the collected surfactants diluted to 40 μL) for the P, CP, and BR extracts averaged for both fuel moisture contents to determine the influence of fuel-bed composition were 38.2, 34.4, and 36.5 mN m^–1^ for the ENVI-18 extracts and 58.1, 48.0, and 42.6 mN m^–1^ for the ENVI-Carb extracts, respectively (averages for individual conditions are shown in Figure 2). The surface tension minimums are low across all fuel-bed types, indicating the presence of strong surfactants in each. Further comparisons of the surface tension minimums cannot be done due to the variability in the total collected particle mass for each experimental condition.

To take into account the moles of surfactant collected from each experiment and the resulting surface tension, we compared CMC values. The calculated CMC values were higher for the organics extracted using the ENVI-18 cartridge from the BBA from the CP and P fuel-bed types than from the BBA of the BR fuel-bed type (Table 1). The surface tension isotherm of the organics extracted from the BBA from P-Wild with the ENVI-18 cartridge was not conducive to determining a CMC (Figure 1). Differences in CMC values of organic extracts between ecoregions could be attributed to structural differences such as polar headgroup, length of the alkyl chain, and charge of surfactants emitted from the combustion of different fuel-bed compositions.^31^

The surface tension isotherms (Figure 1) highlight possible differences in surfactant partitioning at different concentrations as well as possible differences in the strengths of the surfactants. Comparing the surface tension isotherms of the organics extracted from BBA from each fuel-bed type, the Rx curves are all shifted to lower concentrations compared to the Wild curves (Figure 1). For the CP and P curves, this indicates that a higher FRE_norm_ (higher combustion temperatures), under the Wild conditions, produces relatively weaker surfactants. However, the opposite is demonstrated for the BR curves since the Wild condition had lower FRE_norm_ but is shifted to higher surfactant concentrations compared to the BR–Rx curve. Differences in properties such as structure of surfactants produced from the different combustion conditions could alter the CMC and shape of the isotherms and indicate differences in the surfactant production mechanism or emissions.

#### 4.1.2 Variability in Surfactant H/C and O/C due to Fuel Moisture Content

**Figure 4:**
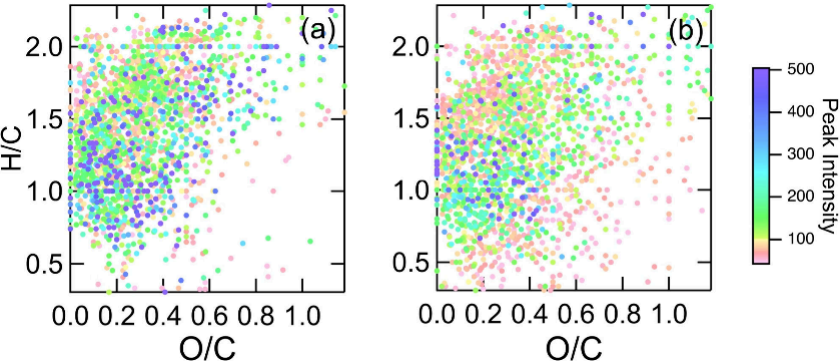
Van Krevelen diagrams showing the H/C and O/C of the formulas identified in the organic fraction extracted from aerosol particles produced from the Coastal Plain (CP) fuel ecoregion with (a) Wild and (b) Rx fuel moisture conditions. Organics were extracted using ENVI-18 cartridges and analyzed in the negative ionization mode. Markers are colored by peak intensity.

The effect of fuel moisture content on the composition of surfactant-like organics extracted from fresh BBA was investigated using mass spectrometric analysis for the CP ecoregion fuel. The van Krevelen diagrams (Figure 4) were used to broadly classify formulas based on their hydrogen to carbon (H/C) and oxygen to carbon (O/C) ratios.

In the negative ionization mode, targeting anionic and nonionic surfactants, the average peak intensities were higher for organics extracted from BBA from the Wild condition compared to those from the Rx condition (Figure S5). Additionally, in the negative ionization mode, the average *m*/*z* of formulas identified in the organic extracts from the BBA from the Wild condition were higher (609 *m*/*z*) than those of the Rx condition (552 *m*/*z*) (Figure S4). A similar trend is observed in the ENVI-Carb extracts, analyzed with positive ionization to target cationic surfactants, with the average peak intensities being higher for the Wild extracts compared to the Rx. However, the average *m*/*z* values are higher in the Rx (663 *m*/*z*) than in the Wild (622 *m*/*z*). Together, this may indicate that more large organics were emitted with the BBA from the Wild condition, showing that different classes of surfactants can be emitted due to different combustion conditions resulting from fuel moisture contents. However, the difference in the peak intensities could also be attributed to differences in ionization efficiencies for specific molecules, which were not investigated in this untargeted analysis. While the peak in *m*/*z* was different based on the ionization technique, both measured large molecules and showed high *m*/*z* values for extracts from BBA from both fuel moisture conditions.

Overall, for both the Wild and Rx, the organic extracts had low O/C (mean <0.37) and high H/C (mean >1.34) (Table 2), consistent with previously identified surface-active compounds (H/C mean >0.40 and O/C mean <0.35) that are aliphatic-like (H/C mean >0.1 and DBE/C < 0.3).^55,84,85^ HULIS and other organic compounds extracted from biomass burning aerosols have previously been found to fall into a similar O/C region (0.1 < O/C < 0.7) and similar H/C region (0.3 < H/C < 2.0)^86^ depending on solubility compared to the O/C and H/C measured here and compared to surfactant-like compounds.

**Table 2:** O/C, H/C, Ratio of Organic Mass to Organic Carbon (OM/OC), DBE/C, Percent Condensed Aromatic (% cond. arom.), Percent Aliphatic (% aliph.), Percent Aromatic (% arom.), Percent HULIS-like (% HULIS-like), and Percent Surfactant-like (% surf-like) Measured with Mass Spectrometry for Organics Extracted Using ENVI-18 (and Ionized with Negative Electrospray Ionization (ESI−)) and ENVI-Carb (and Ionized with Positive Electrospray Ionization (ESI+)) Cartridges from BBA from CP-Wild and CP-Rx Fuel Beds

	CP-Wild		CP-Rx	
	ESI+	ESI–	ESI+	ESI–
O/C	0.37 ± 0.26	0.33 ± 0.22	0.34 ± 0.22	0.34 ± 0.25
H/C	1.35 ± 0.56	1.47 ± 0.40	1.47 ± 0.48	1.34 ± 0.44
OM/OC	1.78 ± 0.42	1.72 ± 0.35	1.74 ± 0.37	1.75 ± 0.40
DBE/C	0.40 ± 0.29	0.34 ± 0.20	0.40 ± 0.29	0.41 ± 0.22
% cond. arom.	22	5	12	12
% aliph.	48	48	55	35
% arom.	11	4	6	7
% HULIS-like	33	37	29	45
% surf-like	54	60	57	55

The organics extracted from the BBA from the Wild condition exhibited the same O/C but higher H/C ratios compared to the Rx in the negative ionization mode (Table 2). This could indicate the presence of more aliphatic and surfactant-like compounds in the BBA from the Rx condition. This may also indicate that different combustion conditions resulting from different fuel moisture contents lead to formation of different classes of organic molecules.

The percentage of aliphatic, aromatic, HULIS, and surfactant-like compounds were calculated from the H/C and O/C ratios (Table 2).^51,87−89^ To determine the contribution of soot or condensed aromatic hydrocarbons to the identified compounds in the organic extracts, the ratio of the double bond equivalent to carbon (DBE/C) was calculated (Table 2). Previous studies have utilized a DBE/C greater than 0.7 as a threshold to signify the presence of condensed aromatic ring structures.^69,90^ For the ENVI-18 extractions, 5% and 12% of the formulas contained DBE/C values indicative of the presence of a condensed aromatic ring for the organics produced during the Wild and Rx fire moisture conditions, respectively, while for the ENVI-Carb extractions, 22% and 12% of the formulas were considered to have condensed aromatic ring structures, respectively. This small fraction of the total formulas containing DBE/C > 0.7 is consistent with that of HULIS targeted samples from ambient biomass burning particles.^69^ Additionally, the small fraction of formulas observed to have condensed aromatic ring structures may be due to the low extraction and ionization efficiencies of aromatic compounds using this method.

Consistent with the O/C and H/C ratios, the organics from BBA produced from the Wild moisture contents showed a higher percentage of aliphatic (48%) and surfactant-like (60%) formulas identified and a lower percentage of aromatics (4%) and HULIS (37%) in the negative ionization mode, compared to the extracts from the Rx moisture contents (35% aliphatic, 55% surfactant-like, 7% aromatic, and 45% HULIS-like (Table 2). The higher percent of surfactant-like molecules from the Wild moisture content (Table 2) are consistent with the higher corresponding surfactant concentration from the Wild moisture content (Figure 3). This further demonstrates the influence of fuel moisture content on the composition of the resulting BBA and the abundance of surfactants in the BBA.

Combined with the surfactant fraction of total particle mass, the comparison of the organic molecular signatures in the organic extracts of the BBA from the CP-Wild and CP-Rx indicates that the combustion conditions, resulting from the fuel moisture contents, play a role in the amount and type of surfactants that are emitted as BBA. The higher FRE_norm_ (combustion temperatures) in the CP-Wild produced a larger surfactant fraction of total particle mass than the CP-Rx, as well as larger H/C ratios, observed in the negative ionization mode.

#### 4.1.3 Changes to Surfactants Due to Photochemical Aging of BBA

To examine the effect of atmospheric aging on the BBA surfactant composition and physical properties, BBA produced from the CP ecoregion fuel were collected following oxidation through the PAM OFR for both moisture conditions. Aging contributed to an overall increase in the surfactant fraction of the total particle mass, from 3.1% to 7.8% and from 0.9% to 3.4% for the Wild and Rx fuel moisture contents, respectively (Figure 3). Here, the assumed molecular weight of 300 g mol^–1^ was used to calculate the mass of surfactants from the moles of surfactant for both the fresh and aged BBA. It is possible that the molecular weight of the surfactants was different in the fresh and aged BBA, which would alter the calculated surfactant mass fractions. However, using a consistent molecular weight allows for a direct comparison between the fresh and aged BBA.

The general increase in the surfactant fraction observed due to aging of BBA is consistent with the SOA enhancement from particle aging. There was a 38% increase in particle mass in the aged BBA from CP-Rx and a 27% increase in particle mass in the aged BBA from the CP-Wild. This increase in overall particle mass suggests the formation of SOA, a large fraction of which may be surfactant-like. The surfactant mass fraction in the aged BBA was within the range but on the higher end of values previously reported in atmospheric aerosols (1.5 to 7%).^28^ This could indicate a mixture of primary and secondary surfactants in atmospheric aerosol particles. Additionally, the difference in SOA production from CP-Rx and CP-Wild is consistent with the larger increase in the surfactant fraction for CP-Rx (278% increase) compared to CP-Wild (152% increase). Additional mechanisms, such as the secondary production of surface-active organics through soot oxidation^91,92^ or primary production through degradation of biomass, may have also increased surfactant fractions in the aged particles.^93^

The surface tension minimums (Figure 2) for the organics extracted using the ENVI-18 cartridge from aged BBA from the CP-Wild and CP-Rx moisture conditions were 43.5 and 43.1 mN m^–1^. These minimum surface tensions are higher than those from the fresh BBA from the CP-Wild and CP-Rx ecoregions without aging (35.4 ± 1.1 and 34.3 ± 0.4 mN m^–1^, respectively). This increase in the surface tension minimums is likely due to the smaller total particle mass collected for the aged BBA and, thus, the smaller total surfactant mass collected (Table 1). The amount of surfactant contributing to the minimum surface tensions, which exclude any UV–vis based extraction and quantification efficiency, were 24 nmol from CP-Wild and 14 nmol from CP-Rx for the aged BBA and 43 nmol from CP-Wild and 28 nmol from CP-Rx for the fresh BBA. The minimum surface tensions of the aged BBA are still significantly depressed compared to that of pure water, even with the smaller number of surfactant moles collected and measured than in the fresh BBA.

Comparison of the surface tension isotherms includes both the measurements of surface tension and the moles of collected surfactants. Figure 1 shows a shift in both surface tension isotherms of the organics extracted from the aged BBA from CP from lower surfactant concentrations to higher. The organics extracted from the aged BBA require larger concentrations to reduce the surface tension of pure water to 55 mN m^–1^, compared to the organics extracted from the fresh BBA (Figure 1). This indicates that the surfactants in aged and fresh BBA have different strengths and compositions.

Previous studies have shown that the effect of photochemical aging on surface tension depression and surfactant concentrations is variable depending on the biomass fuel and exposure time.^18,93^ Giordano et al.^18^ observed a decrease in surface tension depression for surfactants extracted from aerosol from BBA using Manzanita fuels due to longer periods of aging, but observed an increase with aging when using chamise fuels.^18^ Additionally, measured concentrations of methylene blue active substances (anionic surfactants) and ethyl violet active substances (cationic surfactants) from a humic acid solution over a period of 12 h after exposure to ozone showed an increase in surfactant concentrations in the first hour, a decrease after the second hour, and the original levels by the third hour.^93^ They observed continual increases in surfactant concentration when the solution was exposed to natural sunlight.^93^ This indicates that oxidation leads to an increase in the concentration of surfactants in BBA, until degradation begins to occur and may vary based on the oxidation mechanism. This threshold may differ between burn conditions and oxidants used, which is reflected in the variability seen in previous studies. Increasing our understanding of this time scale of formation and degradation of surfactants within BBA is important for understanding their fate and impact on climate effects.

Taken together, the surfactant mass fractions and surface tension isotherms of organics extracted from aged and fresh BBA indicate that aging of BBA may be producing surfactants, but they are different in strength and composition than those directly emitted as BBA. Here, BBA particles were aged in the PAM OFR using OH oxidation, which may not represent all of the aging mechanisms for BBA in the atmosphere. Further investigations of the composition of the surfactants in aged and fresh BBA, as well as additional mechanisms for aging, should be done.

### 4.2 Atmospheric Implications

Freshly emitted aerosol particles produced from combustion of fuels from different ecoregions and moisture contents, resulting in different combustion conditions, contained surfactant fraction averages ranging from 0.9% to 3.1% of the total particle mass. Previous work has shown surfactant fractions of total atmospheric particle mass to be 1.5 to 7%,^27,28^ indicating that the surfactants observed in this study, in BBA from the combustion of fuel beds from three different ecoregions, under two different moisture content conditions, may be potential contributors to surfactants in atmospheric aerosols. Organic compounds extracted from BBA from all fuel beds showed low surface tension minimums (<45 mN m^–1^), indicative of strong surfactants. The mass fraction of surfactants in these BBA can lead to significant reductions in surface tension and potentially impact the CCN activity of BBA. The presence of surfactants in BBA may affect the particle hygroscopicity and contribute to lower surface tensions, depending on the composition of the surfactants and particles, as well as their size.^94,95^ Together, these can contribute to the ability of BBA particles to act as CCN.

Here, we also investigated the surfactant fractions and their properties resulting from combustion of three fuel-bed types, each with two different moisture conditions, to model wildfire (Wild) and prescribed fire (Rx) conditions. Higher surfactant fractions were observed in BBA produced from more efficient combustion as characterized using FRE_norm_, with the highest surfactant fractions in BBA produced from fuel beds that contained only surface fuels from the CP-Wild and P-Wild. The BBA from the BR-Wild conditions had the lowest surfactant fraction, consistent with the lowest FRE_norm_ (longest period of smoldering), caused by the large fraction of duff. This indicates that combustion temperature is an important indicator of surfactant fraction in BBA. The link between combustion conditions and surfactant fraction also indicates that in regions containing only woody and fine fuels, like the P and CP fuel beds, surfactant fractions will be higher from wildfires than prescribed fires. However, if the region contains duff, like with the BR fuel-bed, which does not burn in prescribed fire conditions, the prescribed fires may produce BBA with larger fractions of surfactants than the wildfires in the same regions.

Additionally, particle aging was shown to increase the surfactant fraction of BBA in this study. Aged particles had higher fractions of total particle mass compared to the corresponding freshly emitted particles, indicating that surfactants were produced from photochemical aging of BBA.

Particles with larger fractions of surfactants may be more likely to have low surface tension and thus act as CCN, depending on the complex interactions of particle size and concentration-dependent partitioning of surfactants. Future studies should directly measure the hygroscopicity and CCN potential of BBA from these fuel beds. Future studies should also investigate nonionic surfactants in BBA, which are likely present but were not quantified in this study. Further work on the kinetics of surfactant formation and the mechanisms in which they are formed and degraded during biomass burning events on different time scales and burning conditions will aid in understanding how the changes in chemical and physical properties of BBA will determine their fate and influence in the atmosphere.

## Supporting Information Available

Text describing the measurement and calculations of surfactant extraction efficiencies (Text S1); Table showing the expected moles of surfactants per filter and extraction efficiencies calculated for anionic and cationic surfactant standards (Table S1); Figure showing the measured extraction efficiencies for anionic surfactants (Figure S1); Figure showing the measured extraction efficiencies for cationic surfactants (Figure S2); Figure showing surface tension isotherms for surfactants extracted using the ENVI-Carb cartridges (Figure S3); Figure showing the mass spectra of organics extracted from BBA produced using the CP ecoregion fuel and the Rx and Wild burn conditions and analyzed with negative and positive ionization (Figure S4); Figure showing van Krevelen diagrams of formulas identified in the organic fraction extracted using the ENVI-Carb cartridge from aerosol particles produced from the CP fuel ecoregion under Wild and Rx moisture conditions in the positive mode (Figure S5) (PDF)
