# Supplementary material for: Properties of Surface-Active Organics in Aerosol Particles Produced from Combustion of Biomass Fuels under Simulated Prescribed-Fire and Wildfire Conditions
Source: ACS EST Air. 2025 Jan 3;2(2):264–76. doi: 10.1021/acsestair.4c00243 (PMC11833767; doi:10.1021/acsestair.4c00243)
Supplement: Supplementary file 1 — ea4c00243_si_001.pdf [file ea4c00243_si_001.pdf]

Supporting Information for

Properties of surface-active organics in aerosol particles produced from combustion of biomass fuel under simulated prescribed-fire and wildfire conditions

Ariana M. Deegan[1], Chase K. Glenn[2], Omar El Hajj[2], Anita Anosike[2], Kruthika Kumar[2], Muhammad Abdurrahman[2], Bin Bai[3], Pengfei Liu[3], Joseph O'Brien[4], Rawad Saleh[2], Amanda A. Frossard[1,*]

1. Department of Chemistry, University of Georgia, Athens, GA, 30602, USA
2. School of Environmental, Civil, Agricultural and Mechanical Engineering, University of Georgia, Athens, Georgia 30602, USA
3. School of Earth and Atmospheric Sciences, Georgia Institute of Technology, Atlanta, Georgia, 30602, USA
4. U.S. Department of Agriculture Forest Service, Athens, Georgia, USA

*Corresponding author: Amanda A. Frossard, afrossard@uga.edu

Number of pages: 5

Number of tables: 1

Number of figures: 5

Text S1. Measurements of Extraction Efficiencies

The final determined extraction efficiencies were 63.2 ± 13.9% and 19.3 ± 3.0% for the surfactants extracted using the (i) ENVI-18 cartridge and analyzed with anionic dye and (ii) extracted using the ENVI-Carb cartridge and analyzed with cationic dye, respectively. Known masses of surfactant standards were pipetted onto combusted quartz fiber filters. The filters were then extracted using the same methods described in Section 2.3. The absorbance of the surfactant-dye complex was measured at the corresponding wavelength and applied to the respective class calibration curve to determine a concentration in mM. This was then converted to moles of surfactant per filter and compared to the expected moles of surfactant per filter based on the initial mass of the standards.

Sodium dodecyl sulfate (SDS) and dioctyl sulfosuccinate sodium salt (AOT) were used to determine the extraction efficiency of anionic surfactants, and Hyamine and cetyl triammonium chloride (CTAC) were used to determine the extraction efficiency of cationic surfactants. Three concentrations were measured for the anionic surfactants due to the larger range of expected anionic surfactant concentrations in the samples. Each concentration had a duplicate to determine reproducibility. The two standards for each concentration are referred to as 1st and 2nd duplicate.

Table S1. Calculated extraction efficiencies for the anionic and cationic surfactants at their expected moles.

Sample	Expected Moles on Filter	Extraction Efficiency (%)
SDS 1	4.33*10-9	72
AOT 1	3.98*10-9	63
SDS 2 & 3	3.75*10-9	55
AOT 2 & 3	1.71*10-8	53
Hyamine	3.75*10-9	17
CTAC	3.75*10-9	22

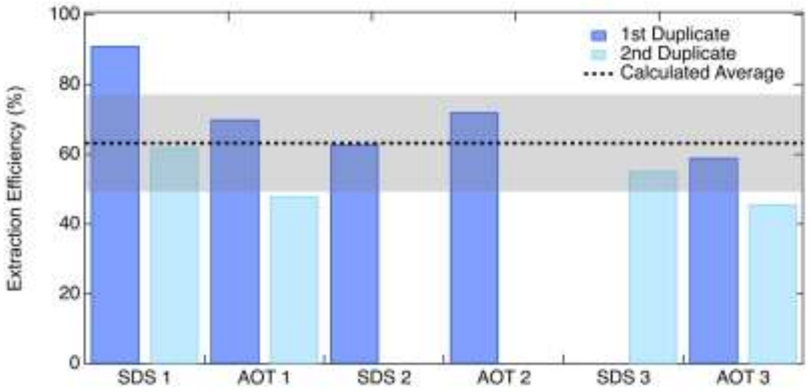

Figure S1. The calculated extraction efficiencies for the three concentrations of anionic standard surfactants (SDS and AOT) measured with colorimetry and UV-Vis spectroscopy. The expected concentrations and calculated extraction efficiencies are shown in Table S1. Each experiment except the second SDS and second AOT experiments was performed in duplicate. The extraction efficiency of the first and second duplicate are shown in dark blue and light blue, respectively. The average extraction efficiency of all measurements is shown as the dashed black line with the standard deviation shown with grey shading.

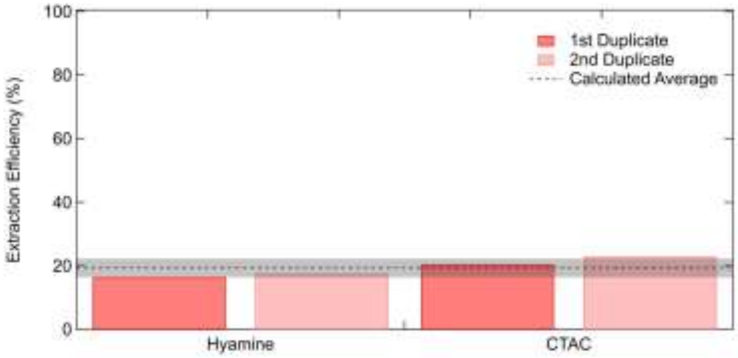

Figure S2. The calculated extraction efficiencies for the two concentrations of cationic standard surfactants (CTAC and Hyamine) measured with colorimetry and UV-Vis spectroscopy. The expected concentrations and calculated extraction efficiencies are shown in Table S1. Each experiment was performed in duplicate. The extraction efficiency of the first and second duplicate is shown in red and light pink, respectively. The average extraction efficiency of all measurements is shown in the dashed black line with the standard deviation shown in with grey shading.

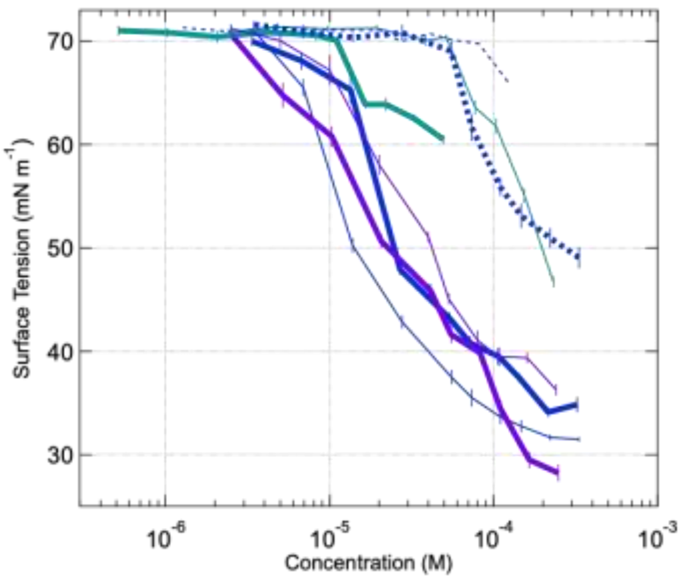

Figure S3. Surface tension isotherms for surfactants extracted using ENVI-Carb cartridges from the aerosol produced from combustion of the three fuel types, Piedmont (P; teal), Coastal Plain (CP; (dark blue), and Blue Ridge (BR; purple) and two fuel moisture contents, Wild (thick lines) and Rx (thin lines). Dashed lines represent surface tension isotherms of surfactant extracts from aged BBA produced from the combustion of the CP fuel beds. Standard deviations shown are from the average of five measurements at each dilution step.

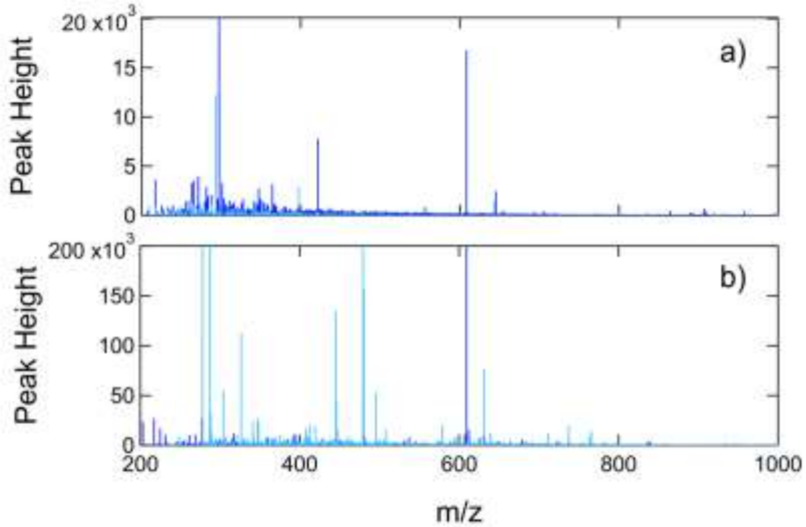

Figure S4. Mass spectra of organic extracts measured with electrospray ionization high resolution mass spectrometry. BBA were produced from CP fuel with Wild (dark blue) and Rx (light blue) fuel moisture conditions. Organics were extracted using (a) an ENVI-18 cartridge and analyzed with negative ionization and (b) an ENVI-Carb cartridge and analyzed with positive ionization.

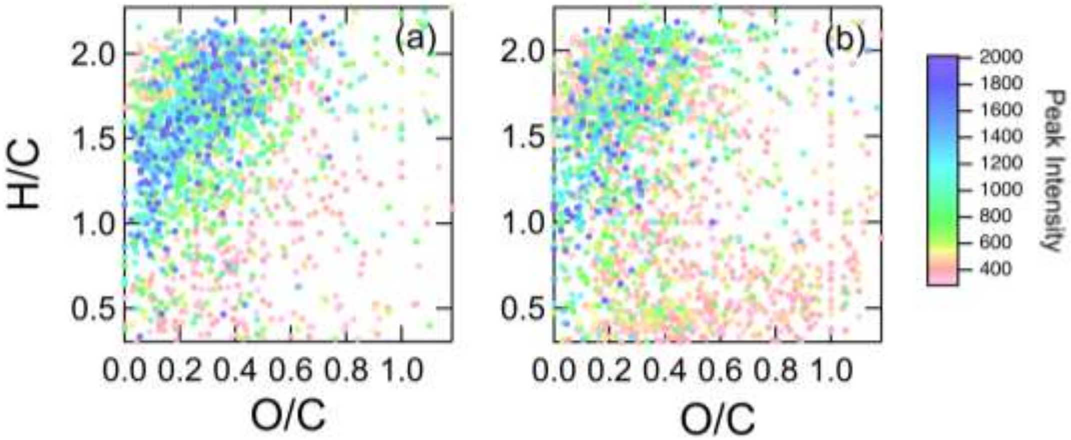

Figure S5. Van Krevelen diagrams showing the H/C and O/C of the formulas identified in the organic fraction extracted from aerosol particles produced from the Coastal Plain (CP) fuel ecoregion with (a) Wild and (b) Rx fuel moisture conditions. Organics were extracted using ENVI-Carb and analyzed in the positive ionization mode. Markers are colored by peak intensity.
